# Author Response to: “Ketamine as Monotherapy in Difficult Airways Is Not Ready for Prime Time”

**DOI:** 10.5811/westjem.2019.9.45175

**Published:** 2019-10-17

**Authors:** Andrew H. Merelman, Michael C. Perlmutter, Reuben J. Strayer

**Affiliations:** * Rocky Vista University College of Osteopathic Medicine, Parker, Colorado; † University of Minnesota Medical School, Minneapolis, Minnesota; ‡ North Memorial Health Ambulance and AirCare, Brooklyn Center, Minnesota; § Maimonides Medical Center, Department of Emergency Medicine, Brooklyn, New York

In reply:

We appreciate the response to our manuscript “Alternatives to Rapid Sequence Intubation: Contemporary Airway Management with Ketamine*”* and value the authors’ perspectives, both competing and complementary.

We agree that flexible endoscopy is a powerful, safety-preserving airway management modality that should be a foundational component of the emergency physician’s arsenal and that, ideally, all emergency physicians would be competent in this skill and use it regularly for fully *awake* intubation technique, facilitated by “meticulous topical anesthesia,” as well as dissociated ketamine-only breathing intubation (KOBI). At the moment, however, the majority of practicing emergency physicians **are not** able to efficiently apply topical anesthesia dense enough to facilitate a fully awake technique in most patients, and **are not** able to able to efficiently intubate using a flexible endoscope, either because they lack the equipment or the skill set, or both.

Furthermore, even providers capable of performing these techniques may not be able to successfully execute a fully awake technique on critically ill patients, who often require intubation quickly and cannot cooperate with a procedure that involves instrumenting their glottis. The relevant comparison is therefore not between fully awake endoscopic intubation technique and KOBI, because most emergency providers *cannot do* the former, whereas nearly all can do the latter. What the literature cannot at present tell us is how KOBI performs against RSI, in patients thought to be especially vulnerable to the harms of RSI. We took special care to indicate in our manuscript that fully dissociated, non-paralyzed intubation technique using conventional or video laryngoscopy has little evidentiary base. Our goal was to provide guidance to facilitate procedural safety and efficacy, and to encourage future research.

We also make special mention in our manuscript of the concern around vomiting present in any awake technique and recommend strategies for mitigating this risk. However, the comment “Emesis occurs in approximately 5–15% of ketamine administrations in adults, which often leads to aspiration…” is misleading. Ketamine-related vomiting typically occurs “late during the recovery phase,” which is why we do not see unacceptable rates of aspiration around ketamine PSA.^1^ The risks of an a*wake* or *breathing* technique must be weighed against the risks of paralysis and providers must be prepared to manage the most important risks associated with any procedure undertaken, which in this case includes vomiting, muscle rigidity, and intubation failure.

We agree with the authors’ concerns for severely acidemic patients and agree that such a patient who is dissociated with ketamine will likely develop relative hypoventilation that, depending on its duration, could be dangerous. Again, however, the current literature is silent on whether these patients, or which subset of these patients, are more likely to be harmed by KOBI compared to RSI (or any other technique). Ultimately, as always, providers must account for a host of factors, including the degree and danger of the underlying physiologic derangement, anticipated anatomic airway difficulty, patient cooperation, and perhaps most importantly the provider’s capabilities. “The best technique for your patient is usually the technique you’re best at doing.” We look forward to more science and discussion as ketamine-based airway techniques are refined to meet emergency providers’ evolving needs and skills.
